# Supplementary material for: 17β-Estradiol-Induced Conformational Changes of Human Microsomal Triglyceride Transfer Protein: A Computational Molecular Modelling Study
Source: Cells. 2021 Jun 22;10(7):1566. doi: 10.3390/cells10071566 (PMC8304645; doi:10.3390/cells10071566)
Supplement: Supplementary file 1 [file cells-10-01566-s001.zip › cells-1163871-supplementary.pdf]

cells

MDPI

Supplemental Materials

17β-Estradiol-Induced Conformational Changes of Human Microsomal Triglyceride Transfer Protein: A Computational Molecular Modelling Study

Yong Xiao Yang [1], Peng Li [1], Pan Wang [1,2] and Bao Ting Zhu [1,2*]

[1] Shenzhen Key Laboratory of Steroid Drug Discovery and Development, School of Life and Health Sciences, The Chinese University of Hong Kong, Shenzhen, Guangdong 518172, China; yangyongxiao@cuhk.edu.cn (Y.X Y.); lipeng@cuhk.edu.cn (P.L.); wangpan@cuhk.edu.cn (P.W.)

[2] Shenzhen Bay Laboratory, Shenzhen 518055, China

* Correspondence: BTZhu@cuhk.edu.cn; Tel.: +086-755-84273851

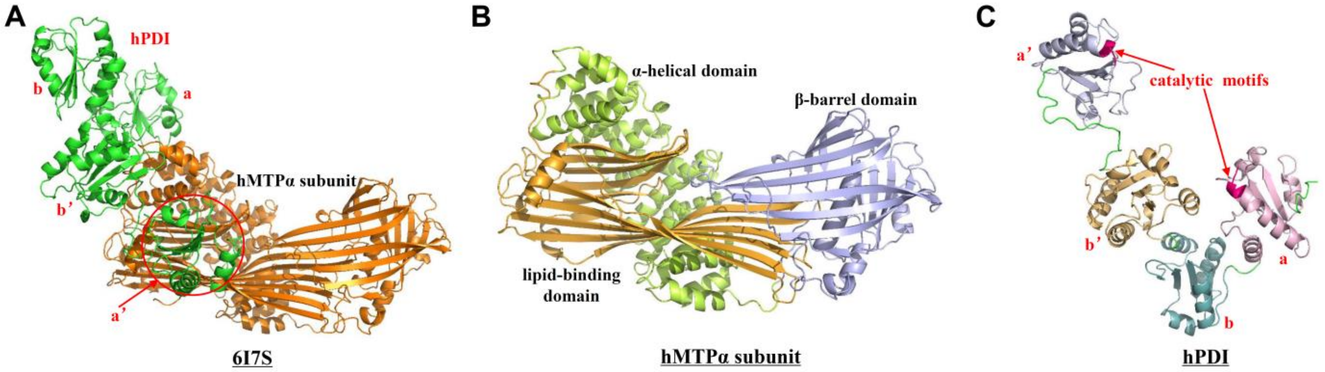

Figure S1. Experimental structure of hMTP complex determined by Biterova *et al.* [1]. **(A)** Crystal structure of hMTP complex (PDB code: 6I7S). The overall structure presents an extended cradle-like conformation composed of hMTPα subunit and hPDI which are colored in orange and green, respectively. **(B)** Structure of hMTPα subunit in hMTP complex. The three domains of hMTPα subunit, β-barrel domain (residues 19–297), α-helical domain (residues 298–603) and lipid-binding domain (residues 604–894) are colored in light blue, limon and bright orange, respectively. **(C)** Structure of hPDI in complex. The four main domains in hPDI, *a* (residues 26–133), *b* (residues 137–232), *b′* (residues 235–349) and *a′* (residues 369–479) domains are colored in light pink, light teal, light orange and blue white, respectively. The two catalytic CXXC motifs (CYS53–GLY54–HIS55–CYS56 and CYS397–GLY398–HIS399–CYS400) are colored in hot pink.

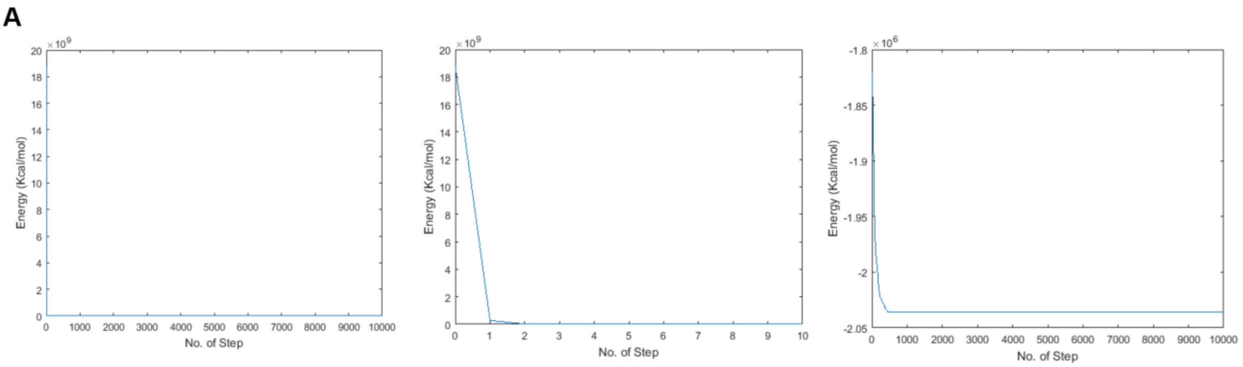

Energy minimization (10000 steps) **first 10 steps** **last 9990 steps**

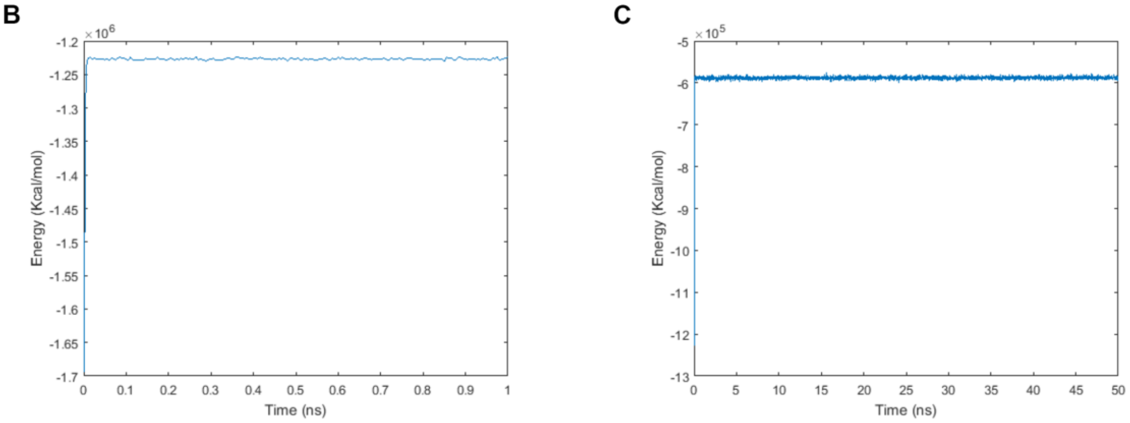

Equilibrium simulation (1 ns) **Production simulation (50 ns)**

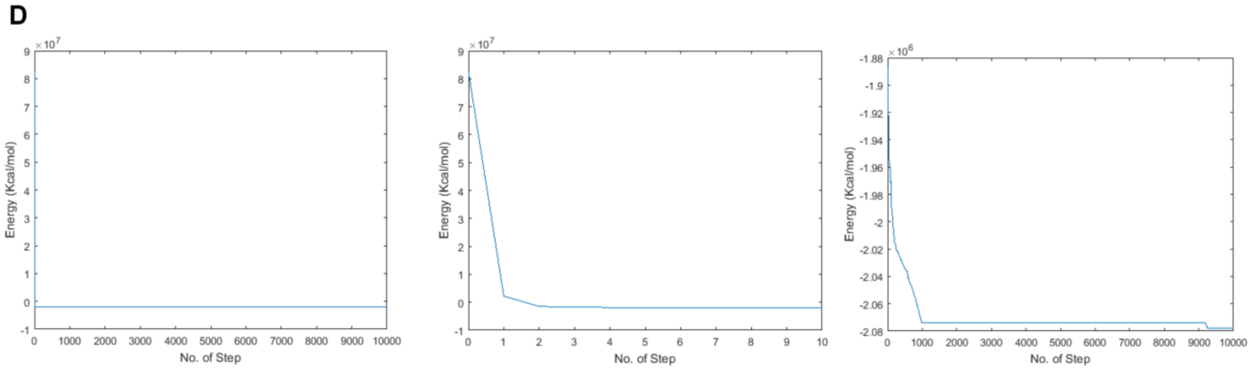

Energy minimization (10000 steps) **first 10 steps** **last 9990 steps**

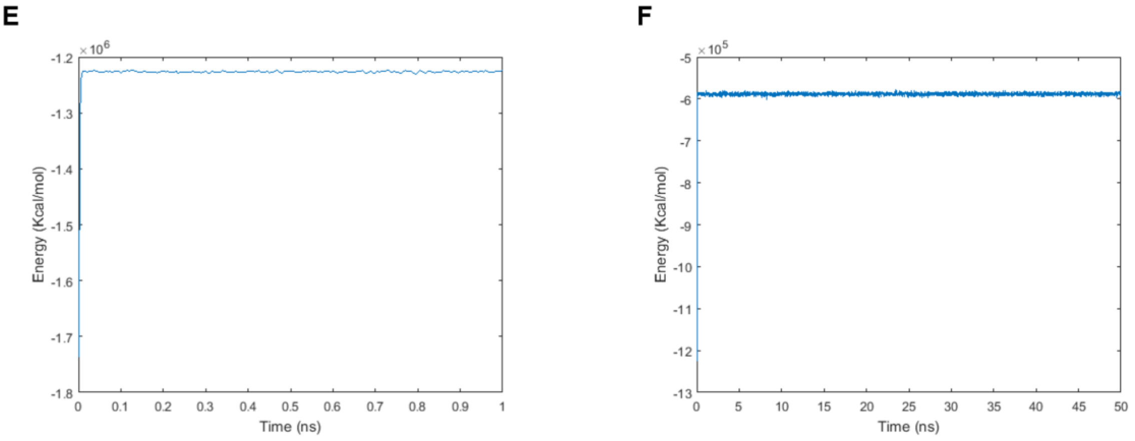

Equilibrium simulation (1 ns) **Production simulation (50 ns)**

G

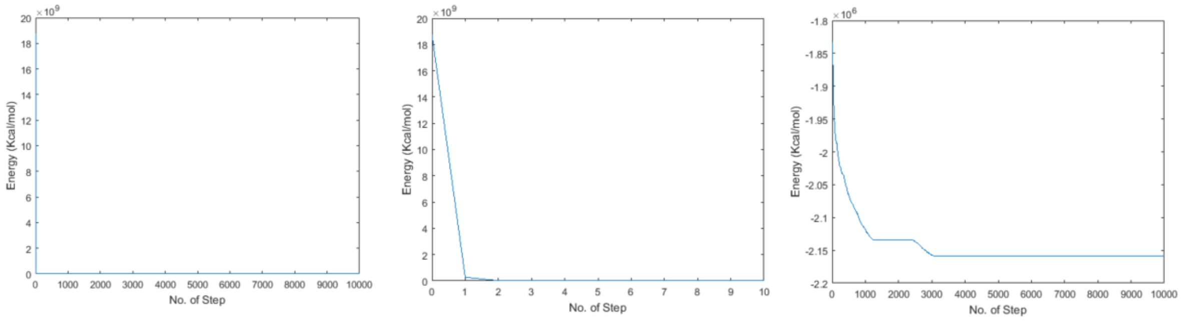

Energy minimization (10000 steps) **first 10 steps** **last 9990 steps**

H

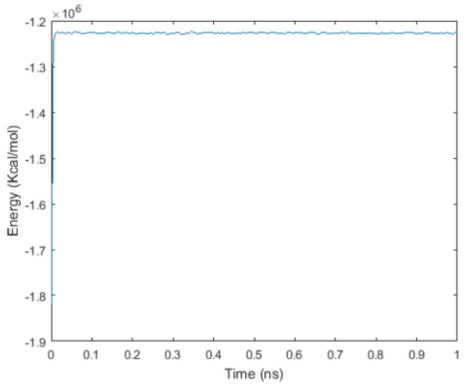

Equilibrium simulation (1 ns)

I

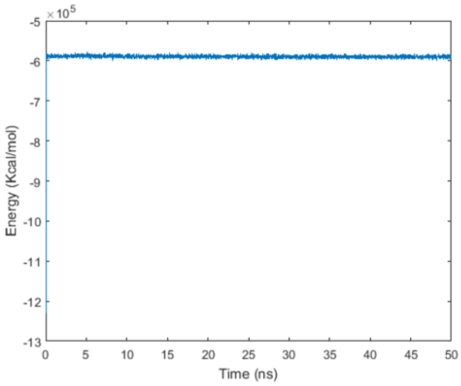

Production simulation (50 ns)

J

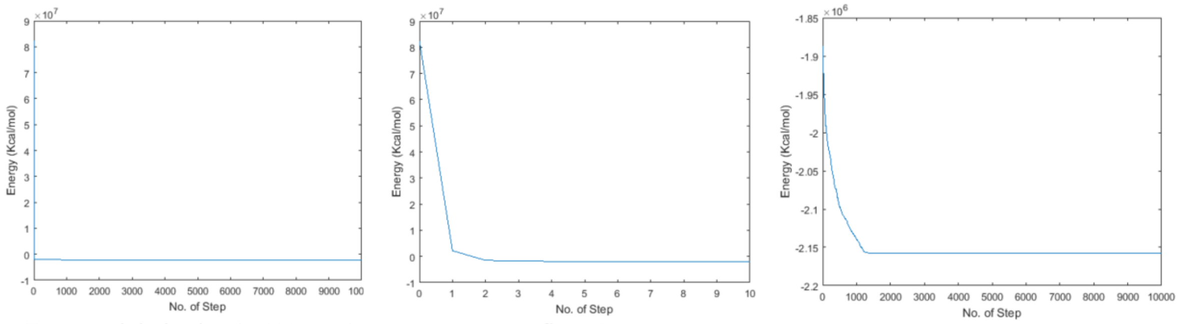

Energy minimization (10000 steps) **first 10 steps** **last 9990 steps**

K

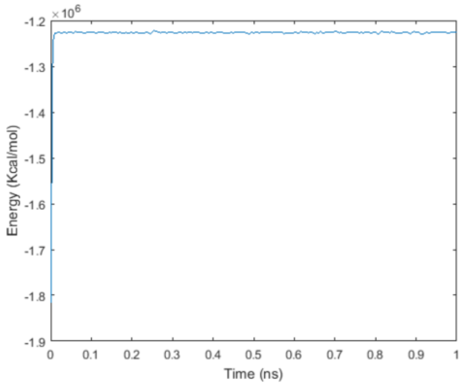

Equilibrium simulation (1 ns)

L

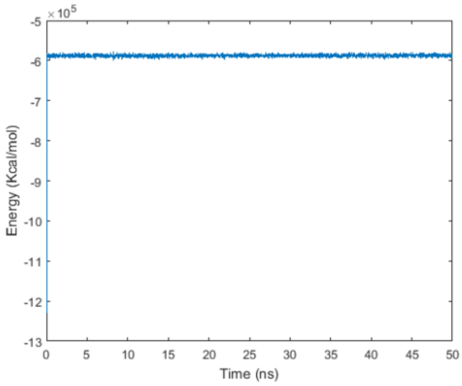

Production simulation (50 ns)

Figure S2. Energy changes during the MD simulation process for hMTP and hMTP–E_2 complexes with/without a hydrogen bond constraint. MD simulations are conducted for all the systems according the procedure: energy minimization for 1000 steps, equilibrium simulation for 1 ns and production simulation for 50 ns. **(A)** Energy minimization for 10000 steps. The left, middle and right panel are energy changes in the process of energy minimization for the total 10000, first 10 and last 9990 steps, respectively. The system energy firstly decreases, then fluctuates around a stable level. The whole system is optimized as much as possible. **(B-C)** Equilibrium simulation for 1 ns and production simulation for 50 ns. **(D-F)** Energy changes during the process of MD simulations for hMTP–E_2 complex with a hydrogen bond constraint. **(G-I)** Energy changes during the process of MD simulations for hMTP complex without the hydrogen bond constraint. **(J–L)** Energy changes during the process of MD simulations for hMTP–E_2 complex without the hydrogen bond constraint. In the process of energy minimization, the system energies firstly decrease, then fluctuate around a stable level. The whole systems are optimized as much as possible. In the process of equilibrium and production simulations, the system energies firstly increase, then fluctuate around a stable level. The whole systems are in dynamic equilibrium states.

Table S1. Number of the interface atom pairs between E_2 and hPDI or hMTPα subunit in the two conformations of hMTP–E_2 complex after MD simulations.

**Interacting region in hPDI or hMTPα subunit**	**Number of the interface atom pairs (cutoff = 5 Å)**	
	hMTP–E_2 complex with a hydrogen bond constraint in the simulation process	hMTP–E_2 complex without the hydrogen bond constraint in the simulation process
b′ domain of hPDI	694	852
α-helical domain of hMTPα subunit	125	77
lipid-binding domain of hMTPα subunit	195	124
All	1014	1053

Table S2. Interface contacts with high mean square fluctuations of distance (MSFD) (higher than 14 Å^2) during the production simulation process.

**No. of interface contact**	**ResID**		**Area of the interface regions (Å^2)**			
	hPDI	hMTPα subunit	With a hydrogen bond constraint in the simulation		Without the hydrogen bond constraint in the simulation	
			hMTP complex	hMTP–E_2 complex	hMTP complex	hMTP–E_2 complex
1	310	888	124.57	28.15	85.94	13.45
2	310	889	126.65	30.50	85.34	17.59
3	310	890	104.22	27.19	64.84	17.25
4	311	889	117.59	32.92	80.13	12.34
5	396	883	3.16	14.73	8.14	10.85
6	396	884	7.30	20.15	14.24	10.49
7	396	885	12.57	23.75	13.54	11.11
8	397	883	3.14	15.84	9.85	11.10

Table S3. Number of interface contacts with difference values of mean square fluctuations of distance (MSFD) between hMTP and hMTP–E2 complexes during the production simulation process (difference value = MSFD of an interface contact in the hMTP–E2 complex – MSFD of the same interface contact in the hMTP complex).

**Interface region**	**Number of interface contacts**					
	With a hydrogen bond constraint			Without the hydrogen bond constraint		
	Absolute difference value of MSFDs (Å^2) ∈ [0, 1]	Difference value of MSFDs (Å^2) ∈ (-7, -1)	Difference value of MSFDs (Å^2) ∈ (1, 7)	Absolute difference value of MSFDs (Å2) ∈ [0, 1]	Difference value of MSFDs (Å^2) ∈ (-11, -1)	Difference value of MSFDs (Å^2) ∈ (1, 11)
**Region 1** (between *a* domain of hPDI and hMTPα)	5	7	11	3	15	5
**Region 2** (between *b′* domain of hPDI and hMTPα)	8	12	7	1	0	26
**Region 3** (between *a′* domain of hPDI and hMTPα)	4	2	21	11	6	10
**Region 3′** (between *C*-terminal of hPDI and hMTPα)	3	4	1	2	5	1
**All**	20	25	40	17	26	42

Table S4. Average and standard deviation of mean square fluctuations of distance (MSFD) in the different interface regions during the production simulation process.

**Interface region**	**Average and standard deviation of mean square fluctuations of distance (MSFD) (Å^2)**							
	With a hydrogen bond constraint				Without the hydrogen bond constraint			
	hMTP complex		hMTP–E_2 complex		hMTP complex		hMTP–E_2 complex	
	Average	Std.	Average	Std.	Average	Std.	Average	Std.
**Region 1** (between *a* domain of hPDI and hMTPα)	2.92	1.87	2.96	1.43	5.29	3.23	3.01	1.65
**Region 2** (between *b′* domain of hPDI and hMTPα)	2.70	1.85	2.14	1.56	0.60	0.58	3.70	2.25
**Region 3** (between *a′* domain of hPDI and hMTPα)	1.28	2.03	3.20	2.15	2.08	0.98	3.61	3.72
**Region 3′** (between *C*-terminal of hPDI and hMTPα)	3.96	2.88	2.38	1.72	3.53	1.56	2.17	1.28
All	2.43	2.16	2.72	1.78	2.61	2.60	3.34	2.63

References

1. Biterova EI, Isupov MN, Keegan RM, Lebedev AA, Sohail AA, Liaqat I, Alanen HI, *et al.* The crystal structure of human microsomal triglyceride transfer protein. Proc Natl Acad Sci U S A 2019, 116: 17251-17260
